# Effects of a pineapple (*Ananas comosus* L.) cannery by-product on growth performance and carcass characteristics in finishing Hanwoo steers

**DOI:** 10.5713/ajas.20.0234

**Published:** 2020-07-02

**Authors:** Yongjun Choi, Sangrak Lee, Youngjun Na

**Affiliations:** 1 Department of Animal Science and Technology, Konkuk University, Seoul 05029, Korea

**Keywords:** Beef Cattle, By-product, Carcass Trait, Pineapple, Steer

## Abstract

**Objective:**

The aim of this study was to determine the effect of pineapple cannery by-product (PCB) level on the growth performance and carcass characteristics of finishing Hanwoo steers.

**Methods:**

The feeding stage was divided into early and late finishing stages. A total of 60 castrated Hanwoo steers (13.9±0.8 months old, 418.8±36.5 kg initial body weight [BW]) were blocked by initial BW and then randomly allotted into 12 pens (five head/pen). The pens were randomly assigned to control (CONT), low PCB (LPCB), or high PCB (HPCB) treatments. These diets contained 0%, 1.5%, or 3.0% of PCB (on a dry matter [DM] basis; as-fed basis was 0%, 10.6%, or 21.2%), respectively.

**Results:**

For the early finishing stage, body weight gain (BWG) and average daily gain (ADG) of the CONT and LPCB feeding groups were greater (p<0.05) than those of the HPCB feeding group. In addition, there were linear and quadratic effects on BWG and ADG with increasing dietary PCB level (p<0.05). The gain to feed (G:F) ratio tends to quadratically decrease with an increasing PCB level in the early finishing stage (p = 0.076). Growth performances of late finishing stage were not affected by PCB level. The marbling score of the LPCB feeding group was similar to that of the CONT feeding group. However, there was a linear decrease (p< 0.05) in marbling score and quality grade among treatments as PCB was increased in the diet. In the *longissimus* muscle free amino acid profile, histidine composition increased linearly (p<0.05) with an increasing level of PCB.

**Conclusion:**

The level of PCB 1.5% DM in diet can be used for finishing steers without any adverse effects on growth and carcass performances. However, there were some negative effects on growth and carcass performance in the HPCB feeding group.

## INTRODUCTION

Ruminant animals take only a few days to adapt to pineapple (*Ananas comosus* L.) by-products in feeds [1]. Although pineapple by-products have sufficient value as feed ingredient in ruminants [1], pineapple by-products do not meet animal requirements as a single feed source due to their low protein and mineral concentrations [2]. Despite these deficiencies, pineapple by-products have been used as a feed ingredient for ruminant animals in local production regions since before 1905 [1]. Canned pineapple is the main product of the pineapple industry [3], and large amounts of pineapple cannery by-products (PCB) are generated during the cannery processing. Previous studies on feeding PCB to dairy and beef cattle have shown that PCB silage can replace 50% of roughage sources in diets without any adverse effect [4], whereas in local production regions, approximately 30% to 100% of fresh PCB has been practically used as a roughage substitute in beef cattle diet [1]. There was reported that PCB silage can replace up to 60% of corn silage with no adverse effects on the growth and carcass characteristics of bulls [5].

Most of these studies have investigated only the short-term (within 100 days) feeding effects of PCB and have also used the low-performance animals (under 500 kg in final body weight [BW]). Moreover, with the exception of the previous research [5], most of the studies have collected the data using survey methodology instead of performing specifically designed animal experiments. For these reasons, more work is needed to determine the long-term effect of PCB on the growth and carcass characteristics of Hanwoo (*Bos taurus coreanae*) steers. In addition, intramuscular fat is the most important factor determine the quality of beef in developed countries. In South Korea, the feeding stage is generally divided into growing and finishing stages considering the growth characteristics by the aging of cattle. In this point of view, the herd management of the finishing stage is important because it is the major period for intramuscular fat accumulation [6]. Accordingly, the null hypothesis tested in the present study was that increasing the level of PCB would have no effect on growth performance and carcass characteristics in finishing Hanwoo steers.

## MATERIALS AND METHODS

### Animal care

The experiment was carried out in a conventional beef cattle facility at Phocheon, Gyeonggi, Korea (latitude 38.01° and longitude 127.35°). All research protocols were approved by Konkuk University Animal Care and Use Committee (approval number: KU16139).

### Pineapple cannery by-product

The PCB was obtained from Shinsegae Food Processing Facility (Table 1; Icheon, Gyeonggi, Korea). The PCB which used in the current analyses contained the skin, crown ends, bud ends, and cores. The chemical composition of PCB used in this experiment was shown in Table 1 and the composition of lipids and amino acids was presented as Supplementary Table A.1 and A.2.

### Animals and experimental design

The feeding stage was divided into two stages (early and late finishing stages). For each stage, experimental diets were formulated and produced to meet or exceed the Korean Feeding Standard for Hanwoo (*Bos taurus coreanae*) requirements [7] (Table 2). A total of 60 castrated Hanwoo steers were used for the experiment. All steers were vaccinated for foot and mouth disease before the start of the experiment. For the early (13.9±0.8 months old, 418.8±36.5 kg initial BW) and the late (22.8±0.8 months old, 617.2±49.7 kg initial BW) finishing stage, steers were blocked by initial BW and then randomly allotted into 12 pens (five head/pen). The pens were randomly assigned to control (CONT), low PCB (LPCB), or high PCB (HPCB) treatments. The diets used in these treatments contained 0%, 1.5%, or 3.0% (on a dry basis; as-fed basis was 0%, 10.6%, and 21.2% of PCB, respectively). Animals were fed twice a day at 0900 and 1700 h *ad libitum*, with 5% to 10% refusals. The early and late finishing stages lasted for 273 and 110 d, respectively. Offered diets and orts were measured daily for dry matter intake (DMI). Experimental diets and orts were sampled monthly and stored at −20°C for chemical analysis.

### Chemical analysis

All samples were dried and ground to pass through a 1-mm screen (Wiley Mill; Thomas Scientific, Swedesboro, NJ, USA). These samples were analyzed in duplicate for DM (934.01 method), ash (934.01 method), crude protein (CP; 990.03 method), and ether extract (EE; 920.39 method) [8]. Neutral detergent fiber (NDF; 2002.04 method) was analyzed according to Mertens [9] using sodium sulfite and heat-stable α-amylase (Sigma A3306; Sigma Chemical CO., St. Louis, MO, USA). Acid detergent fiber (ADF; 973.18) was measured according to the methods of Van Soest et al [10]. An ANKOM fiber analyzer 200 (ANKOM Technology Crop., Macedon, NY, USA) was used for NDF and ADF analyses. The non-fiber carbohydrate (NFC) content of feeds was calculated by subtraction of CP, NDF, EE, and ash from 100.

### Blood sampling and analysis

Blood samples were collected at d 253 (about 22.3±0.8 month old) and 360 (about 25.9±0.8 month old) after starting the experiment. Blood samples (15 mL) were collected via the jugular vein using 18-gauge needles and transferred to silicon-coated serum tubes (Vacutainer, BD, Franklin Lakes, NJ, USA). Serum and plasma were separated by centrifugation at 2,000×*g* at 4°C for 20 min. Serum samples were stored at −80°C and were subsequently analyzed using an automated chemical analyzer (Model 7180 Clinical Analyzer, Hitachi Ltd, Tokyo, Japan) following the instructions for metabolite and mineral analyses in the manufacturer’s manual. Reagents for analyses of albumin, glutamic-oxaloacetic transaminase, glutamic pyruvate transaminase, blood urea nitrogen (BUN), creatine, triglycerides, high-density lipoproteins, low-density lipoproteins, calcium, inorganic phosphorus, magnesium, and non-esterified fatty acids were purchased from JW Medical (Seoul, Korea).

### Carcass characteristics

As the last step of the experiment, all animals were transported to a commercial slaughterhouse (NH Meat Processing Facility, Bucheon, Korea) and slaughtered after 24 h of rest. At 24-h postmortem, carcass characteristics, including carcass weight, back-fat thickness, marbling score, *longissimus* muscle (LM) area, dressing percentage, and quality grade, were determined following the guidelines of the Animal Products Grading Service, Korea [11]. A Minolta Chroma Meter (CR-400, Minolta Inc., Tokyo, Japan) was used for measuring the lightness (L*), redness (a*), and yellowness (b*) of LM tissue. Meat color was determined in triplicate. Approximately 300 g of LM tissue samples were collected from all steers and were stored with vacuum packing at −80°C until subsequent fatty acid (FA), and free amino acid (FAA) analyses.

### Fatty acid composition analysis

For FA analysis, lipids in freeze-dried LM samples were extracted using a 2:1 (v/v) chloroform:methanol solvent [12] and then methylated [13]. After methylation, the supernatant was injected into a gas chromatograph (Agilent 7890A, NY, USA) equipped with a flame ionization detector and a capillary column (60 m×0.25 mm×0.25 μm; DB-23, Agilent, USA) operated at 50°C for 1 min, then programmed at 8°C/min to 215°C, and held at this temperature for 10 min [14]. The standard solution was Supelco 37 Component FAME Mix (Sigma-Aldrich Inc., St. Louis, MO, USA).

### Free amino acid composition analysis

To determine concentrations of FAAs in LM, each sample was placed in a volumetric flask to which was added 75% methanol. The flask was then placed into an ultrasonic bath for 60 min at 30°C. The extracts were then passed through a 0.2-μm filter. For subsequent analysis, HPLC (Ultimate 3000, Thermo Fisher Scientific Inc., Waltham, MA, USA) was used as described by Henderson et al [15]. The FAA composition was quantified on the basis of known amounts of standards (Agilent 5061-3330, USA).

### Statistical analysis

Data were analyzed using SAS PROC MIXED (Version 9.4, SAS Institute Inc., Cary, NC, USA) [16]. The model included PCB level as the fixed effect and initial BW as the random effect. Least squares means among treatments were compared using the PDIFF option when treatment effect was significant. Orthogonal contrast for linear and quadratic effects was performed. Treatment effects were considered significant at p< 0.05, and trends were considered at 0.05≤p<0.10.

## RESULTS

### Nutrient compositions

Dry matter, ash, CP, EE, ADF, NDF, NFC, and WSC concentrations in PCB were 9.2%, 4.9%, 5.7%, 1.2%, 17.7%, 39.4%, 48.8%, and 5.4% DM basis, respectively (Table 1). Values for the TDN of CONT, LPCB, and HPCG were 71.0%, 71.3%, and 71.6% DM in the early finishing stage, respectively, and those of late finishing stage were 75.3%, 75.4%, and 75.6% DM, respectively (Table 2). The CP concentration of CONT, LPCB, and HPCB was similar in each finishing stage (Table 2). In the experimental feed, as the concentration of PBC increased, those of fibrous material increased, and those of NFC decreased (Table 2).

### Body weight gain, feed intake, and gain to feed ratio

For the early finishing stage, the body weight gain (BWG) and average daily gain (ADG) of the CONT and LPCB feeding groups were greater (p<0.05) than those of the HPCB feeding group (Table 3). There were linear and quadratic effects on BWG and ADG with increasing dietary PCB level (p<0.05). The gain to feed (G:F) ratio tends to quadratically decrease as increasing PCB level in the early finishing stage (p = 0.076). Growth performances in the late finishing stage were not affected by PCB level.

### Blood profiles

For the early finishing stage, albumin concentration increased linearly (p<0.05) with increasing dietary PCB level and BUN concentration tended to decrease linearly (p<0.10) with increasing dietary PCB level (Table 4). For the early finishing stage, inorganic P concentration quadratically changed (p< 0.05) with increasing dietary PCB level and Mg concentration was increased (p<0.05) with increasing dietary PCB level (Table 4).

The serum triglyceride concentration of late finishing steers increased linearly (p<0.05) with increasing dietary PCB level.

### Carcass traits and meat color

The marbling score of the LPCB feeding group was similar to that of the CONT feeding group (Table 5). However, there was a linear decrease (p<0.05) in marbling score and quality grade among treatments as PCB level in the diet increased. Meat color of LPCB was higher than those of other treatments (p<0.05). The texture was linearly increased (p<0.05) with increasing PCB level. For the quality grade, the percentage of 1^+^ grade quadratically decreased (p<0.05) with increasing PCB level, then the percentage of 2 grade linearly increased (p<0.05) with increasing PCB level. The sum of 1^+^ and 1^++^ grade was greater LPCB treatment than those of other treatments (Table 5).

Increasing PCB level also tended to be associated with a linear decrease (p<0.10) in redness (a*) of LM muscle (Table 6).

### *Longissimus* muscle fatty acid and amino acid profiles

There was no significant difference in the LM FA profiles (Table 7). For the LM FAA profiles, histidine composition increased linearly (p<0.05) with an increasing level of PCB (Table 8). Alanine tends to increase quadratically with increasing PCB level and phenylalanine tends to decrease quadratically with increasing PCB level.

## DISCUSSION

In general, the Korean beef cattle industry adopts a three-stage feeding system (growing, early finishing, and late finishing) for beef steers [7]. The primary objective of the growing stage is to maximize the development of bone, organs, and the gastrointestinal tract of steers for subsequent fattening. The nutrient composition of the early finishing diets in the current experiment was similar among treatments, there were linear and quadratic decreases in BWG and ADG as well as increases in G:F ratio with an increasing level of dietary PCB, even though DMI was significantly did not differ among treatments. In the current experiment, HPCB (3.0% DM) feeding resulted in adverse effects on the growth performance of early finishing stage steers and carcass traits. It, therefore, appears that long-term feeding of high levels of PCB negatively affects growth performance, whereas short-term usage does not have any adverse effects.

Growth performance and adipose tissue development status during the early finishing stage influence the subsequent weight gain and intermuscular fat deposition of animals [7]. Therefore, the adverse effects in early finishing stage indicate the possibility of retardation of intramuscular fat deposition. In contrast, there were no effects on the growth performance of the late finishing steers. It appears that because of the lower BWG and ADG as well as lower G:F ratio of the late finishing steers compared with the early finishing steers, the overall potential negative effect of PCB on growth performance might be diminished in the late finishing stage. In general, BWG and ADG were decreased during the late finishing stage, even though DMI and energy concentrate were similar to those in the growing or early finishing stage, because steers require greater maintenance energy during the late finishing stage [7]. There was reported that pineapple by-product can replace 50% of roughage sources in dairy cattle ration without any adverse effect [4]. There was reported that the DM, organic matter (OM), and metabolizable energy intake decreased linearly when pineapple by-product silage was gradually increased (0% to 31% DM) in the diet as a substitute for corn silage, whereas BWG and ADG were not affected by the level of pineapple by-product silage [5]. However, it is difficult to directly compare the findings of this study with those of the current study because Prado et al [5] used lower performance animals (under 500 kg in final BW) than those used in the present study. In addition, pineapple by-products used in the study of Prado et al [5] have different characteristics because they used the ensiled pineapple by-product.

There was a quadratic decrease in BUN concentration with an increasing level of PCB in both the growing and early finishing stages. The concentration of BUN reflects the quantity of nitrogen absorbed from the rumen and small intestine because it is a pool with input from urea synthesis by renal and hepatic tissues [17,18]. The concentration of BUN has been used as a useful indicator of the protein metabolism of ruminants [19,20]. When unused ammonia enters the portal blood, it is transported to the liver where it is converted into urea. The urea then circulates in the blood and reaches the kidneys where it is excreted with the urine or diffuses from the blood into the rumen, saliva, or mammary glands [21]. Pineapple stems and fruit are rich in the proteolytic enzyme bromelain [22]. There is evidence to indicate that this protease has high hydrolytic activity on feed ingredients such as fish meal, soybean meal, and maize gluten meal [23]. Therefore, it is possible that the action of bromelain contained in PCB might increase protein degradability in the gastrointestinal tract, as a result of degrading the indigestible protein contents in mixed rations. In addition, the BUN concentration is highly correlated with milk urea nitrogen (MUN) concentration [24,25], high BUN or MUN concentration can decrease the conception rates of lactating cows and heifers [26–28]. Although the current study was conducted using beef cattle, there is potential for PCB supplementation in animal diets to improve the fertility of cows.

In ruminant, triglyceride concentration in the blood depends on the concentrate feed level in the feed [29]. In this study, as an increasing dietary PCB level in experimental feed, the ratio of NFC and TDN increased. For these reasons, it seems that it has affected the concentration of triglyceride content in the blood. In the animal body, inorganic P indicates play a role in energy production, bone, and muscle growth and Mg has a role in energy production, muscle shrink, nerve function, and maintain bone [30]. In this study, although there were significant differ inorganic P and Mg content in blood among the treatments, those of level were considered that it did not seem to affect the health of animals during the entire experimental period.

As PCB level increased, the marbling score and quality grade showed a linear decrease. There was no significant difference in other carcass traits. There was reported that DM and CP intake of adult sheep was linearly increased with increasing of dehydrated pineapple by-product (0%, 3.5%, 7.0%, 10.5%, and 14%) [31]. Furthermore, when dehydrated pineapple by-product was replaced (0% to 100%) with coast cross hay (*Cynodon dactylon*) in goat diets, the apparent nutrients digestibility of goats was linearly increased without any decline in body weight or feed intake [32,33]. This study showed the opposite result, it considered that was affected by the amount of pineapple juice in the experimental feed. Pineapple juice was reported that showed pH 3.3 to 3.6 [34], low pH of feed ingredient has an effect on decreasing of the rumen pH, and it causes a decrease in OM intake in a ruminant animal [35]. In this study, although the moisture contents of each experimental diet were same by adding the tap water, the experimental feed contained a large amount of pineapple juice compared with the control diet, it considered that has negative effects on feed intake and carcass traits. Furthermore, it appears that the depression of growth performance by low DM intake during the early finishing stage could affect intramuscular fat deposition. Because a large amount of adipose tissue is developed in early finishing steers [36], growth performance during the early finishing stage can influence subsequent weight gain and fat deposition in beef cattle [7]. As meat color was determined using criteria made by lightness, redness, and yellowness content of meat [11], it means that the change of meat color has the same direction as those of redness value. Also, in the meat color, redness value has the biggest role in determining the meat color value. In this result, although increasing of dietary PCB level had an effect on the decrease of redness in the meat, it is not affects final quality grade [11].

There was no significant difference among the treatments in the fatty acid composition except for C17:1. In this study, it wasn’t found any direct evidence, dietary PCB supplementation has an effect on the change of fatty acid composition in LM. Furthermore, the magnitude of the C17:1 composition change was negligible level. Certain FAA and peptides have been shown to influence meat flavor and human gastrointestinal function [37]. In the current experiment, only the free histidine composition of LM increased linearly with an increasing level of PCB. Erickson et al [38] reported that histidine inhibits ferric iron-catalyzed enzymatic and non-enzymatic lipid peroxidation in membranes. Moreover, β-alanyl-l-histidine and β-alanyl-l-1-methylhistidine have anti-oxidant properties in cells [39]. Apparently, therefore, increasing the level of PCB in beef cattle diets can increase the free histidine composition of LM, thereby improving the functionality of beef for humans.

## CONCLUSION

The PCB levels of 1.5% DM in the diet can be used for finishing steers without any adverse effects on growth and carcass performances. However, there were some negative effects on growth and carcass performance when dietary levels of PCB were increased to 3.0% DM. There was a linear increase in LM free histidine composition with an increase in the PCB content of beef cattle ration. Therefore, PCB levels of approximately 1.5% DM can be used in beef cattle ration without adverse effects. Moreover, PCB can promote an increase the free histidine composition of LM, thereby improving the functionality of beef for humans.

## Figures and Tables

**Table 1:** Chemical composition of pineapple cannery by-product

Chemical composition	Pineapple cannery by-product
DM (%)	9.2
Ash (% DM)	4.9
CP (% DM)	5.7
EE (% DM)	1.2
NDF (% DM)	39.4
ADF (% DM)	17.7
NFC (% DM)	48.8
WSC (% DM)	5.4

DM, dry matter; CP, crude protein; EE, ether extract; NDF, neutral detergent fiber; ADF, acid detergent fiber; NFC, non-fiber carbohydrate; WSC, water soluble carbohydrate.

**Table 2:** Ingredients and chemical composition of experimental diets

Items	Early finishing stage1)			Late finishing stage1)		
	
	CONT	LPCB	HPCB	CONT	LPCB	HPCB
Ingredients (% DM)						
Pineapple cannery by-product	0.0	1.5	3.0	0.0	1.5	3.0
Corn, cracked	9.2	10.1	11.0	26.3	25.9	25.4
Protein supplement	8.9	9.6	10.3	14.0	14.0	14.0
Commercial concentrate 1, pelleted	45.3	43.4	41.5	-	-	-
Commercial concentrate 2, pelleted	-	-	-	30.0	30.0	30.0
Almond hull	6.0	6.7	7.5	9.1	8.4	7.6
Brewer’s grain	4.1	3.6	3.0	3.0	3.0	3.0
Beet pulp	7.2	7.1	6.9	-	-	-
Tall fescue, hay	4.6	4.0	3.3	-	-	-
Alfalfa pellet	1.5	1.4	1.3	4.0	4.0	4.0
Rice straw	13.2	12.7	12.3	13.6	13.3	13.0
Chemical composition						
DM (%)	65.1	65.2	65.2	65.1	65.1	65.1
Ash (% DM)	6.6	6.6	6.5	6.4	6.4	6.4
CP (% DM)	14.6	14.5	14.5	14.0	14.0	14.0
EE (% DM)	2.8	2.8	2.8	3.2	3.2	3.2
NDF (% DM)	44.3	43.5	42.7	36.8	36.6	36.5
ADF (% DM)	23.8	23.5	23.1	19.1	18.9	18.7
NFC (% DM)	31.7	32.6	33.5	39.6	39.8	40.0
TDN (% DM)	71.0	71.3	71.6	75.3	75.4	75.6

DM, dry matter; CP, crude protein; EE, ether extract; NDF, neutral detergent fiber; ADF, acid detergent fiber; NFC, non-fiber carbohydrate; TDN, total digestible nutrients.

1) CONT, control; LPCB, low pineapple cannery by-product (1.5% DM); HPCB, high pineapple cannery by-product (3.0% DM).

**Table 3:** Effects of pineapple cannery by-product on dry matter intake, body weight, average daily gain, and gain to feed ratio of finishing Hanwoo steers

Items	Level of PCB1)			SEM	p-value	
	
	CONT	LPCB	HPCB		Linear	Quadratic
Early finishing						
Initial BW (kg)	422.8	416.7	416.8	29.9	0.707	0.825
Final BW (kg)	628.6	626.8	593.3	26.2	0.079	0.327
BWG (kg)	205.7^a^	210.0^a^	176.5^b^	6.5	0.008	0.030
ADG (kg/d)	0.76^a^	0.77^a^	0.65^b^	0.02	0.009	0.041
DMI (kg/d)	9.25	9.23	9.09	0.20	0.208	0.618
G:F ratio	0.083	0.085	0.075	0.6	0.067	0.076
Late finishing						
Initial BW (kg)	628.6	626.8	593.3	26.2	0.079	0.327
Final BW (kg)	667.7	668.9	633.1	13.7	0.107	0.299
BWG (kg)	44.6	37.7	39.8	3.6	0.368	0.333
ADG (kg/d)	0.42	0.36	0.38	0.04	0.453	0.370
DMI (kg/d)	9.60	9.39	9.22	0.30	0.233	0.955
G:F ratio	0.044	0.038	0.041	0.004	0.622	0.376

SEM, standard error of the mean; BW, body weight; BWG, body weight gain; ADG, average daily gain; DMI, dry matter intake; G:F, gain to feed; DM, dry matter.

1) CONT, control; LPCB, low pineapple cannery by-product (1.5% DM); HPCB, high pineapple cannery by-product (3.0% DM).

ab Means in same row with different superscripts differ significantly (p<0.05).

**Table 4:** Effects of pineapple cannery by-product on serum blood parameters of early finishing Hanwoo steers

Items	Level of PCB1)			SEM	p-value	
	
	CONT	LPCB	HPCB		Linear	Quadratic
Early finishing						
Albumin (g/dL)	3.6^b^	3.7^ab^	3.7^a^	0.05	0.016	0.517
GOT (U/L)	57.0	60.5	64.1	3.52	0.186	0.989
GPT (U/L)	22.6	22.6	22.3	0.77	0.716	0.833
BUN (mg/dL)	19.3^a^	20.1^a^	17.4^b^	0.66	0.068	0.046
Creatine (mg/dL)	1.4	1.5	1.4	0.05	0.698	0.437
Triglycerides (mg/dL)	16.9	17.1	18.3	0.67	0.168	0.592
HDL (mg/dL)	222.0	229.3	247.5	14.0	0.152	0.704
LDL (mg/dL)	65.4	62.9	74.5	5.68	0.286	0.337
Inorganic P (mg/dL)	7.3^b^	7.9^a^	7.3^b^	0.16	0.868	0.012
Mg (mg/dL)	2.8^b^	2.9^b^	3.0^a^	0.05	0.026	0.292
NEFA (mmol/L)	152.0	174.7	192.9	36.0	0.439	0.959
Late finishing						
Albumin (g/dL)	3.7	3.8	3.8	0.05	0.489	0.782
GOT (U/L)	66.9	71.6	73.4	2.92	0.145	0.689
GPT (U/L)	23.8	23.5	23.5	0.76	0.795	0.830
BUN (mg/dL)	22.4	21.6	20.8	0.63	0.630	0.981
Creatine (mg/dL)	1.4	1.4	1.4	0.04	0.573	0.251
Triglycerides (mg/dL)	20.4^b^	23.8^b^	26.6^a^	1.18	0.005	0.847
HDL (mg/dL)	111.3	105.5	112.2	2.36	0.793	0.058
LDL (mg/dL)	23.5	23.6	21.9	2.04	0.596	0.729
Inorganic P (mg/dL)	7.5	7.8	7.7	0.12	0.271	0.425
Mg (mg/dL)	2.5	2.6	2.6	0.05	0.390	0.668
NEFA (mmol/L)	211.9	218.9	200.0	21.6	0.705	0.637

SEM, standard error of the mean; GOT, glutamic-oxaloacetic transaminase; GPT, glutamic pyruvic transaminase; BUN, blood urea nitrogen; HDL, high-density lipoproteins; LDL, low-density lipoproteins; NEFA, non-esterified fatty acids; DM, dry matter.

1) PCB, pineapple cannery by-product; CONT, control; LPCB, low pineapple cannery by-product (1.5% DM); HPCB, high pineapple cannery by-product (3.0% DM).

ab Means in same row with different superscripts differ significantly (p<0.05).

**Table 5:** Effects of pineapple cannery by-product on carcass traits of finishing Hanwoo steers1)

Items	Level of PCB2)			SEM	p-value	
	
	CONT	LPCB	HPCB		Linear	Quadratic
Back fat (mm)	12.6	11.6	12.7	0.79	0.931	0.307
LM3 area (cm^2^)	84.0	83.9	84.1	2.52	0.978	0.962
Carcass weight (kg)	400.1	393.9	377.6	12.16	0.223	0.742
Dressing (%)	64.9	65.6	64.7	0.50	0.820	0.203
Marbling	5.6^a^	5.7^a^	4.3^b^	0.27	0.009	0.054
Meat color	4.6^b^	4.9^a^	4.6^b^	0.07	1.000	0.009
Fat color	3.0	3.0	3.1	0.03	0.252	0.497
Texture	1.1^b^	1.1^b^	1.4^a^	0.06	0.004	0.148
Maturity	2.1	2.0	2.0	0.06	0.252	0.497
Quality grade3)	3.5^a^	3.6^a^	3.0^b^	0.15	0.041	0.133
1^++^ (%)	10	5	0	4.4	0.143	1.000
1^+^ (%)	35	60	25	9.1	0.458	0.025
1 (%)	50^a^	25^b^	50^a^	7.3	1.000	0.020
2 (%)	5	10	25	5.3	0.025	0.458

SEM, standard error of the mean; LM, *longissimus* muscle.

1) Carcass traits and grade were evaluated according to guidelines of the Animal Products Grading Service, Korea (APGS, 1995).

2) PCB, pineapple cannery by-product; CONT, control; LPCB, low pineapple cannery by-product (1.5% DM); HPCB, high pineapple cannery by-product (3.0% DM).

3) Average of the total quality grade of each pen, 2 grade, 1; 1 grade, 2; 1^+^ grade, 3; 1^++^ grade, 4.

ab Means in same row with different superscripts differ significantly (p<0.05).

**Table 6:** Effects of pineapple cannery by-product on longissimus muscle color of finishing Hanwoo steers

Items	Level of PCB1)			SEM	p-value	
	
	CONT	LPCB	HPCB		Linear	Quadratic
Meat color2)						
L*	39.7	40.0	37.4	1.19	0.158	0.286
a*	17.1	16.2	15.3	0.72	0.097	0.989
b*	11.0	10.7	10.1	0.49	0.195	0.794

SEM, standard error of the mean; DM, dry matter.

1) PCB, pineapple cannery by-product; CONT, control; LPCB, low pineapple cannery by-product (1.5% DM); HPCB, high pineapple cannery by-product (3.0% DM).

2) Meat color expressed according to the Commission Internationale de l’Eclairage (CIE) L* (lightness), a* (redness), and b* (yellowness) format.

**Table 7:** Effects of pineapple cannery by-product on *longissimus* muscle fatty acid profiles (g/100 g) of finishing Hanwoo steers

Items	Level of PCB1)			SEM	p-value	
	
	CONT	LPCB	HPCB		Linear	Quadratic
C4:0	3.17	2.52	2.39	0.68	0.414	0.744
C11:0	0.07	0.06	0.06	0.01	0.535	0.414
C12:0	0.11	0.10	0.11	0.01	0.772	0.496
C14:0	0.02	0.03	0.03	0.01	0.820	0.253
C14:1	4.02	4.14	3.61	0.22	0.223	0.258
C15:0	1.36	1.38	1.19	0.15	0.467	0.589
C15:1	0.25	0.25	0.23	0.01	0.416	0.723
C16:0	30.60	29.88	28.95	0.77	0.161	0.914
C17:0	4.84	5.19	4.51	0.29	0.372	0.129
C17:1	0.60	0.56	0.59	0.01	0.622	0.028
C18:0	10.29	9.99	10.91	0.59	0.261	0.209
C18:1	40.45	41.53	42.76	1.03	0.146	0.956
C18:1n9c	0.14	0.19	0.13	0.02	0.760	0.088
C18:2n6c	0.14	0.16	0.16	0.01	0.373	0.643
C18:3n6	0.07	0.06	0.06	0.01	0.514	0.674
C18:3n3	0.20	0.26	0.23	0.04	0.644	0.380
C20:0	1.99	1.89	2.13	0.15	0.516	0.394
C21:0	0.12	0.10	0.12	0.01	0.804	0.289
c9t11 CLA	0.08	0.08	0.08	0.01	0.709	0.910
t10c12 CLA	0.04	0.04	0.04	0.01	0.913	0.671
C20:2	0.03	0.04	0.04	0.01	0.384	0.345
C20:3n6	0.43	0.55	0.51	0.16	0.707	0.707
C22:1n9	0.25	0.25	0.29	0.04	0.512	0.707
C20:4n6	0.43	0.42	0.54	0.07	0.305	0.529
C23:0	0.25	0.29	0.27	0.09	0.836	0.829
C24:0	0.04	0.03	0.03	0.01	0.421	0.586
C22:6n3	0.03	0.02	0.03	0.00	0.790	0.255
SFA	52.85	51.46	50.70	1.05	0.183	0.809
MUFA	45.32	46.48	47.19	0.85	0.155	0.838
PUFA	1.83	2.07	2.11	0.29	0.516	0.778

SEM, standard error of the mean; CLA, conjugated linoleic acid; SFA, saturated fatty acid; MUFA, mono-unsaturated fatty acid; PUFA, poly-unsaturated fatty acid; DM, dry matter.

1) PCB, pineapple cannery by-product; CONT, control; LPCB, low pineapple cannery by-product (1.5% DM); HPCB, high pineapple cannery by-product (3.0% DM).

**Table 8:** Effects of pineapple cannery by-product on *longissimus* muscle free amino acid composition of growing-finishing Hanwoo steers

Amino acids (μg/g)	Level of PCB1)			SEM	p-value	
	
	CONT	LPCB	HPCB		Linear	Quadratic
Glutamic acid	116.6	110.5	103.8	8.7	0.323	0.973
Asparagine	41.9	39.5	44.3	2.9	0.587	0.336
Serine	100.7	91.3	92.0	6.3	0.357	0.533
Glutamine	827.9	750.5	958.1	87.8	0.322	0.218
Histidine	48.7^b^	53.3^ab^	61.6^a^	3.2	0.019	0.655
Glycine	123.7	132.8	133.5	7.7	0.393	0.663
Threonine	87.9	79.7	85.9	5.5	0.798	0.316
Arginine	139.3	135.8	128.2	10.1	0.457	0.875
Alanine	2,355.8	2,103.2	2,571.0	134.7	0.288	0.057
Taurine	543.5	521.9	609.3	67.3	0.507	0.525
Tyrosine	100.2	86.6	91.8	5.9	0.344	0.225
Valine	119.6	102.4	113.4	8.3	0.610	0.197
Methionine	61.1	51.4	52.7	4.2	0.196	0.310
Phenylalanine	97.2	81.9	93.1	5.6	0.623	0.087
Isoleucine	83.0	73.1	74.2	6.2	0.343	0.491
Leucine	157.2	136.4	140.4	11.0	0.308	0.383
Lysine	110.5	110.4	107.6	7.1	0.781	0.880
Proline	132.4	129.8	143.3	7.1	0.278	0.349

SEM, standard error of the mean; DM, dry matter.

1) PCB, pineapple cannery by-product; CONT, control; LPCB, low pineapple cannery by-product (1.5% DM); HPCB, high pineapple cannery by-product (3.0% DM).

ab Means in same row with different superscripts differ significantly (p<0.05).

## References

[1] Muller ZO (1978). Feeding potential of pineapple waste for cattle. World Anim Rev.

[2] Gowda NKS, Vallesha NC, Awachat VB, Anandan S, Pal DT, Prasad CS (2015). Study on evaluation of silage from pineapple (*Ananas comosus*) fruit residue as livestock feed. Trop Anim Health Prod.

[3] Tran AV (2006). Chemical analysis and pulping study of pineapple crown leaves. Ind Crops Prod.

[4] Sruamsiri S (2007). Agricultural wastes as dairy feed in Chiang Mai. Anim Sci J.

[5] Prado IN, Lallo FH, Zeoula LM, Caldas Neto SF, Nascimento WG, Marques JA (2003). Bulls performance in feedlot with levels of substituting corn silage by pineapple by-products silage. Rev Bras Zootec.

[6] Park SJ, Beak S-H, Jung DJS (2018). Genetic, management, and nutritional factors affecting intramuscular fat deposition in beef cattle - a review. Asian-Australas J Anim Sci.

[7] Rural Development Administration National Institute of Animal Science (2012). Korean feeding standard for Hanwoo.

[8] Horwitz W, Latimer GW (2005). Official methods of analysis of AOAC International.

[9] Mertens DR (2002). Gravimetric determination of amylase-treated neutral detergent fiber in feeds with refluxing in beakers or crucibles: collaborative study. J AOAC Int.

[10] Van Soest PJ, Robertson JB, Lewis BA (1991). Methods for dietary fiber, neutral detergent fiber, and nonstarch polysaccharides in relation to animal nutrition. J Dairy Sci.

[11] Animal Products Grading Service (1995). Report of business for animal products grading.

[12] Folch J, Lees M, Sloane Stanley GH (1957). A simple method for the isolation and purification of total lipides from animal tissues. J Biol Chem.

[13] Lepage G, Roy CC (1986). Direct transesterification of all classes of lipids in a one-step reaction. J Lipid Res.

[14] Garces R, Mancha M (1993). One-step lipid extraction and fatty acid methyl esters preparation from fresh plant tissues. Anal Biochem.

[15] Henderson JW, Ricker RD, Bidlingmeyer BA, Woodward C (2000). Rapid, accurate, sensitive, and reproducible HPLC analysis of amino acids.

[16] SAS Institute Inc (2015). Base SAS 9.4 procedures guide.

[17] Lewis D (1957). Blood-urea concentration in relation to protein utilization in the ruminant. J Agric Sci.

[18] Owens FN, Bergen WG (1983). Nitrogen metabolism of ruminant animals: historical perspective, current understanding and future implications. J Anim Sci.

[19] Preston RL, Byers F, Stevens KR (1978). Estrogenic activity and growth stimulation in steers fed varying protein levels. J Anim Sci.

[20] Hof G, Vervoorn MD, Lenaers PJ, Tamminga S (1997). Milk urea nitrogen as a tool to monitor the protein nutrition of dairy cows. J Dairy Sci.

[21] Hammond AC Update on BUN and MUN as a guide for protein supplementation in cattle.

[22] Chittenden RH, Joslin EP, Meara FS (2018). On the ferments contained in the juice of the pineapple, ananassa sativa, together with some observations on the composition and proteolytic action of the juice.

[23] Lee TT, Chiou WS, Hsu JC, Yu B (2000). Evaluation of protease digestion on various feed proteins by chemical assay. (2) Effects on distribution of polypeptide pattern of hydrolyzed feed proteins. J Chin Soc Anim Sci.

[24] Thornton RF (1970). Factors affecting the urinary excretion of urea nitrogen in cattle. II. The plasma urea nitrogen concentration. Aust J Agric Res.

[25] Hennessy DW, Nolan JV (1988). Nitrogen kinetics in cattle fed a mature subtropical grass hay with and without protein meal supplementation. Aust J Agric Res.

[26] Elrod CC, Van Amburgh M, Butler WR (1993). Alterations of pH in response to increased dietary protein in cattle are unique to the uterus. J Anim Sci.

[27] Elrod CC, Butler WR (1993). Reduction of fertility and alteration of uterine pH in heifers fed excess ruminally degradable protein. J Anim Sci.

[28] Ferguson JD, Galligan DT, Blanchard T, Reeves M (1993). Serum urea nitrogen and conception rate: the usefulness of test information. J Dairy Sci.

[29] Aurousseau B, Bauchart D, Calichon E, Micol D, Priolo A (2004). Effect of grass or concentrate feeding systems and rate of growth on triglyceride and phospholipid and their fatty acids in the *M. longissimus* thoracis of lambs. Meat Sci.

[30] Committee on Nutrient Requirements of Beef Cattle, National Research Council (2016). Nutrient requirements of beef cattle: eighth revised edition.

[31] Ferreira ACH, Neiva JNM, Rodriguez NM, Campos WE, Borges I (2009). Nutritional evaluation of pineapple industry by-product as additive on elephant grass silage. Rev Bras Zootec.

[32] de Carvalho Correia MX, Costa RG, da Silva JHV, de Carvalho FFR, de Medeiros AN (2006). Use of dehydrated pineapple by-product in diets for growing goats: digestibility and performance. Rev Bras Zootec.

[33] Costa RG, Correia MXC, Da Silva JHV, De Medeiros AN, De Carvalho FFR (2007). Effect of different levels of dehydrated pineapple by-products on intake, digestibility and performance of growing goats. Small Rumin Res.

[34] Shamsudin R, Daud WRW, Takriff MS, Hassan O (2007). Physicochemical properties of the josapine variety of pineapple fruit. Int J Food Eng.

[35] Rustomo B, AlZahal O, Odongo NE, Duffield TF, McBride BW (2006). Effects of rumen acid load from feed and forage particle size on ruminal pH and dry matter intake in the lactating dairy cow. J Dairy Sci.

[36] Yamazaki T (1977). Effects of age and fatness on the meat quality and quantity of beef cattle. 1. The growth of various organs and tissues of Japanese Black breed steers.

[37] San Gabriel A, Uneyama H (2013). Amino acid sensing in the gastrointestinal tract. Animo Acids.

[38] Erickson MC, Hultin HO (1992). Influence of histidine on lipid peroxidation in sarcoplasmic reticulum. Arch Biochem Biophys.

[39] Hipkiss AR, Gaunitz F (2014). Inhibition of tumour cell growth by carnosine: some possible mechanisms. Animo Acids.

