# Supplementary material for: Effects of a pineapple (Ananas comosus L.) cannery by-product on growth performance and carcass characteristics in finishing Hanwoo steers
Source: Anim Biosci. 2020 Jul 2;34(2):233–42. doi: 10.5713/ajas.20.0234 (PMC7876721; doi:10.5713/ajas.20.0234)
Supplement: Supplementary file 1 [file ajas-20-0234-suppl.pdf]

Table A.1 Fatty acid composition (g/100g) of pineapple cannery by-product[1]

Fatty acid composition, g/100g	Pineapple cannery by-product
C10:0	0.51
C12:0	1.23
C16:0	22.86
C16:1	4.05
C18:0	12.08
C18:1n9c	21.98
C18:2n6c	18.55
C18:3n3	10.82
C20:0	1.22
C20:1	0.76
C20:4n6	0.50
C22:0	1.07
C22:1n9	0.22
C24:0	4.15
SFA	43.12
UFA	56.88
MUFA	27.01
PUFA	29.87
Ω 3	10.82
Ω 6	19.05
Ω 3 : Ω 6	0.57

[1]SFA, saturated fatty acid; UFA, unsaturated fatty acid; MUFA, monounsaturated fatty acid; PUFA, polyunsaturated fatty acid.

Table A.2. Amino acid composition of pineapple cannery by-product

Amino acids, %	Pineapple cannery by-product
Indispensable AA[1]	
Arginine	4.4
Histidine	2.4
Isoleucine	5.1
Leucine	7.4
Lysine	2.5
Methionine	1.4
Phenylalanine	4.7
Threonine	5.6
Valine	6.2
Total indispensable AA	39.9
Dispensable AA	
Alanine	7.3
Aspartic acid	14.0
Glutamic acid	15.2
Glycine	6.4
Proline	6.0
Serine	7.0
Tyrosine	3.2
GABA[2]	0.9
Total dispensable AA	60.1

[1]AA, amino acid.

[2]GABA, gamma-aminobutyric acid.
